# Then and Now: Investigating Anthropometrics and Child Mortality among Females in Malawi

**DOI:** 10.3390/ijerph19106171

**Published:** 2022-05-19

**Authors:** Sally Sonia Simmons, John Elvis Hagan, Thomas Schack

**Affiliations:** 1 Department of Social Policy, London School of Economics and Political Science, London WC2A 2AE, UK; ssimmons@edu.hse.ru; 2 Institute of Demography, National Research University-Higher School of Economics, 101000 Moscow, Russia; 3 Department of Health, Physical Education & Recreation, College of Education Studies, University of Cape Coast, Cape Coast PMB TF0494, Ghana; 4 Neurocognition and Action Research Group—Biomechanics, Faculty of Psychology & Sport Sciences/CITEC, Bielefeld University, Postfach 10 01 31, 33501 Bielefeld, Germany; thomas.schack@uni-bielefeld.de

**Keywords:** child mortality, females, Malawi, obesity, overweight, underweight

## Abstract

Information on the concentration of body mass index and child death among females in Malawi, where the epidemics of weight gain have been disconcerting and preventable deaths among children linger, is limited. Therefore, the study examined the polarity of body mass index and the death of children among females. Using data from the Malawian Demographic and Health Survey from 2000 to 2015–2016, the study applied for the first time the index of concentration at the extremes and indirect demographic techniques to estimate the polarity of body mass index and child mortality among 65,499 females aged 15 to 49 years. The preponderance of obesity more than doubled from 2000 to 2015–2016 and was highest among females who were older (35–49 years), urban dwellers, rich, and located in districts within the central and southern regions. In addition, child survival was low among underweight, overweight, and obese females. While national-, regional-, and individual-level statistics are in development, these findings provide helpful information for health experts and other stakeholders to initiate appropriate age-region specific programs and interventions in Malawi, including targeting females in the high socio-economic bracket.

## 1. Introduction

The burden of high undernutrition—wasting, stunting, or body mass index (BMI) >18.5 kg/m^2^—and overnutrition (high BMI (≥25 kg/m^2^)) is being exacerbated in Sub-Saharan Africa (SSA) [1]. The epidemics of high BMI, in particular, have been greatest among vulnerable groups, especially females of reproductive age (15–49 years) through to elderly years [2,3]. About 16% and 7% of females in their reproductive years are overweight (BMI ≥ 25–29.9 kg/m^2^) and obese (BMI ≥ 30 kg/m^2^), respectively [4]. In addition, pregnancies, including those to compensate for prior or subsequent child death [5,6,7,8,9] and associated delivery, increase the risk of overweight or obesity [10,11]. As a result, in Eastern African countries such as Malawi, where child mortality and parity among females remain high [12,13,14,15], there is a strong female bias in overweight and obesity [6,8,15]. One in five female Malawians of reproductive age is overweight [16]. Ntenda and Kazambwe [15] also reported that the estimated number of overweight or obese female Malawians more than doubled (10% to 21%) between 1991 and 2017. Moreover, about 50.1 children die per 1000 live births in the country [17]. This poor birth outcome estimate (50.1 children die per 1000 live births) and other pregnancy complications among females aged 15–49 years, according to Nkoka et al. [16], might be related to overweight and obesity.

Among the many factors implicated in the relationship between BMI, poor maternal health, and birth outcomes; age, residence, wealth, education, and behavior are mainstream in Malawi [15,18]. The prevalence of obesity is higher among urban females than in their rural counterparts [19,20]. Furthermore, the risk of overweight and obesity increases with age. In addition, a spectrum of poor maternal outcomes, including infant and neonatal deaths, is prevalent among young (≤20 years) mothers/females who are often underweight, overweight, or obese [21]. Apart from the age and residential disadvantage, Makoka’s [22] study on inequities in health in Malawi also revealed a wealth gradient in health among females of reproductive age. Mndala and Kudale [23], and Nutor et al. [24] expounded on Makoka’s [22] finding by reporting that the epidemics of obesity engulfing Lilongwe, Zomba, and Blantyre instead of other regions in Malawi might be effects of increment in wealth, rapid urbanization, and material conditions in general.

Despite the congruence of scholarship ([3,15,19,25,26,27]) on the covariates of BMI and child mortality in Malawi, empirical evidence on the concentration of BMI at the extremes among females is limited. Besides, no study has assessed the association between females’ BMI—overweight, obese, and underweight—and child mortality in Malawi. In addition, it is uncertain whether the epidemics of BMI have been the results of specific temporal exposure-effect. While research on BMI and child mortality covariates may be relevant, such a study is a parody of the known. The present study, therefore, expanded on previous works [15,22] by using macro-level metrics to capture the dynamics of BMI and child mortality per females’ anthropometric disposition to account for potential health inequalities and guide the implementation of malnutrition (overnutrition and undernutrition) and child mortality prevention or reduction interventions in Malawi. Accordingly, the study investigated the excess proportion of females with either high (obese) or low (underweight) BMI ranked by sociodemographic groups and child mortality among females within different BMI groups.

## 2. Materials and Methods

### 2.1. Data Source

**Figure 1:**
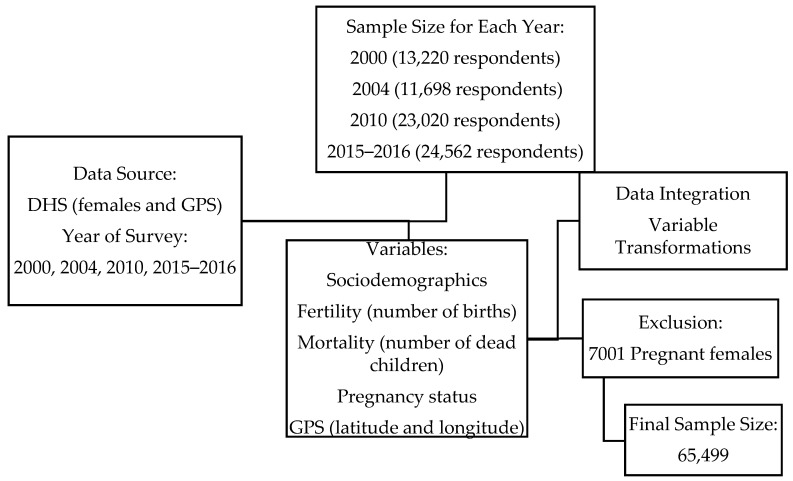
A flowchart outlining data and variable selection. Source: authors’ construct.

Four Standard Malawi Demographic and Health Surveys (MDHS) in 2000, 2004, 2010, and 2015–2016) were the data for the study. These MDHS data were retrieved from DHS. These four MDHSs are comparable, nationally representative, cross-sectional household surveys [28,29,30] that focus on key demographic and health indicators used for the evaluation and surveillance of the population, health, and nutritional programs [31,32,33]. In addition, the data for these years allow for between-group health comparison and assessment of the association between child mortality and BMI to draw possible exposure-effect conclusions [34,35]. The MDHS forms part of broad categories of surveys designed by the DHS program and funded by the United States Agency for International Development (USAID). Each MDHS selected for the study (female and Global Positioning System [GPS]) had a specified number of respondents: 2000 (13,220 respondents); 2004 (11,698 respondents); 2010 (23,020 respondents); 2015–2016 (24,562 respondents) (see Figure 1). The MDHS employed context-specific and standard multistage and geographically stratified probability-based sampling methods for respondent selection [36]. As part of this approach, existing but modified standard enumeration areas (SEAs) were used as primary sampling units (PSUs). A detailed description of the Malawi DHS study methods and materials, including the data and data collection procedures, is provided by the National Statistical Office (NSO) [36,37,38,39].

### 2.2. Study Area

**Figure 2:**
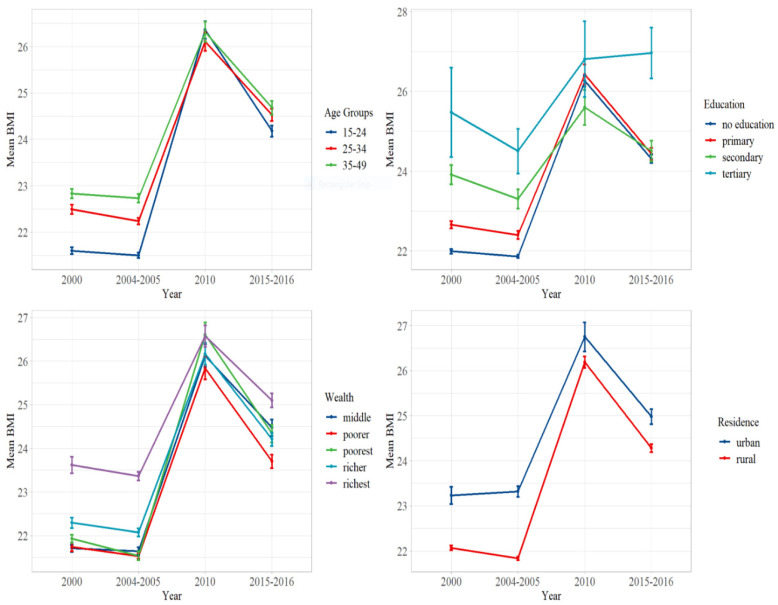
Average body mass index of females in different demographic groups in Malawi. Source: computed from MDHS 2000, 2004, 2010, 2015–2016. Note: BMI: body mass index.

**Figure 3:**
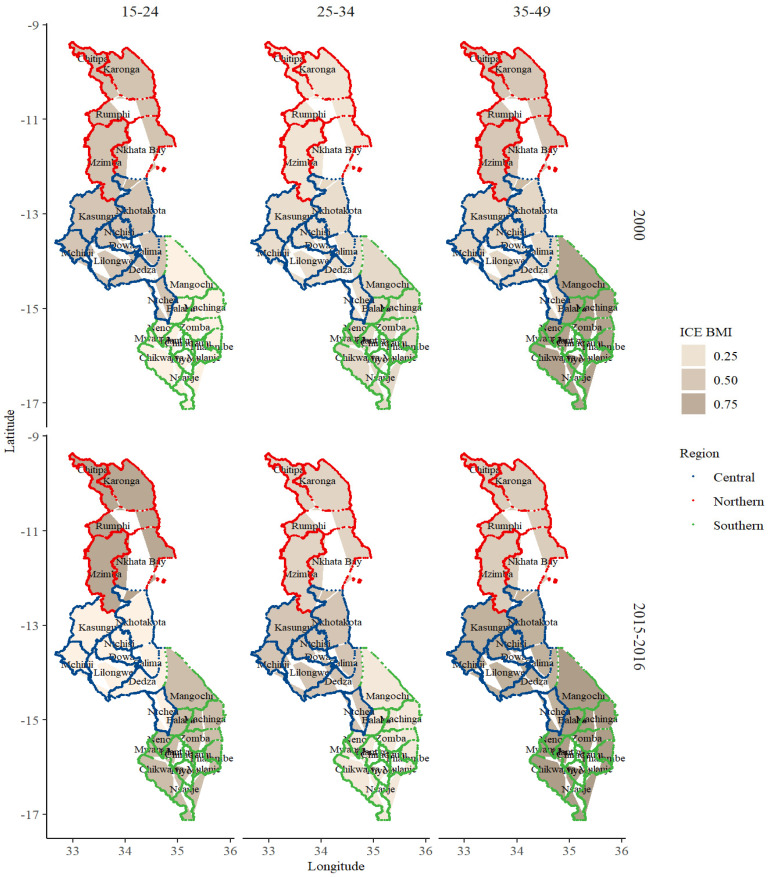

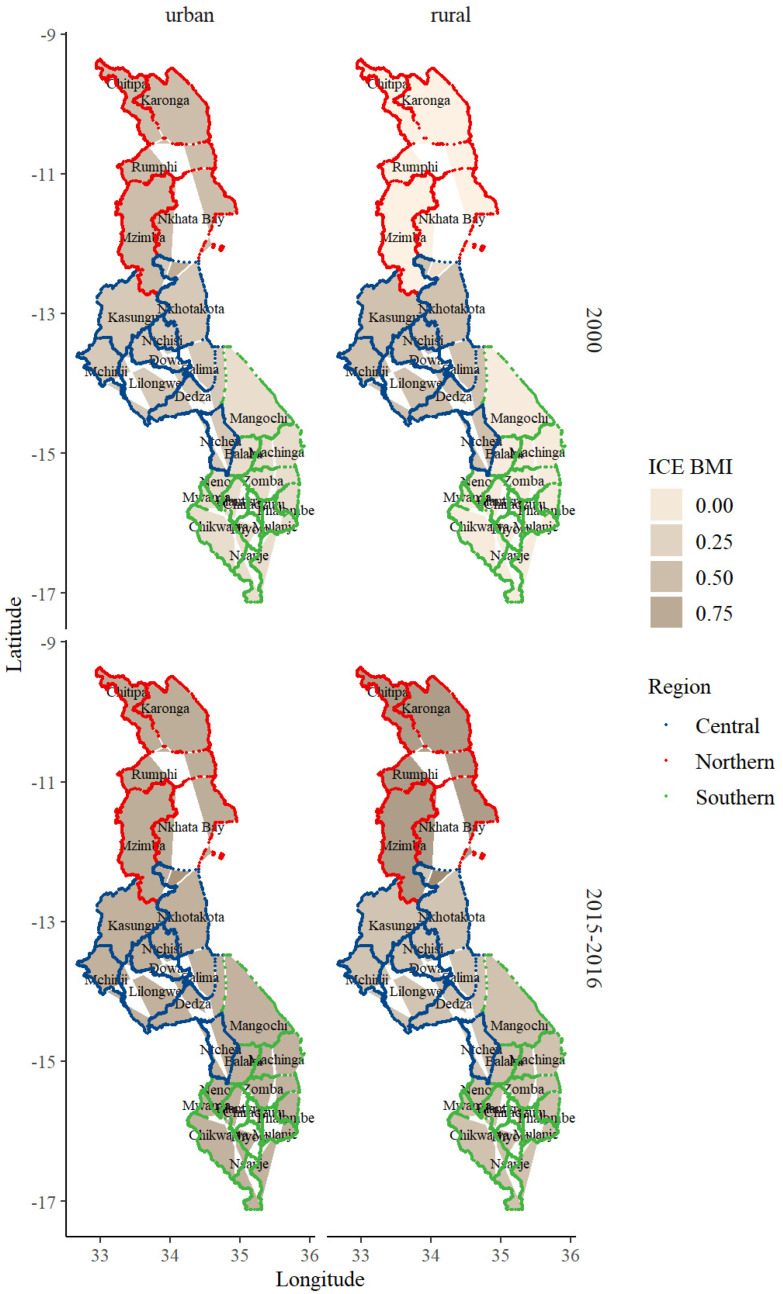

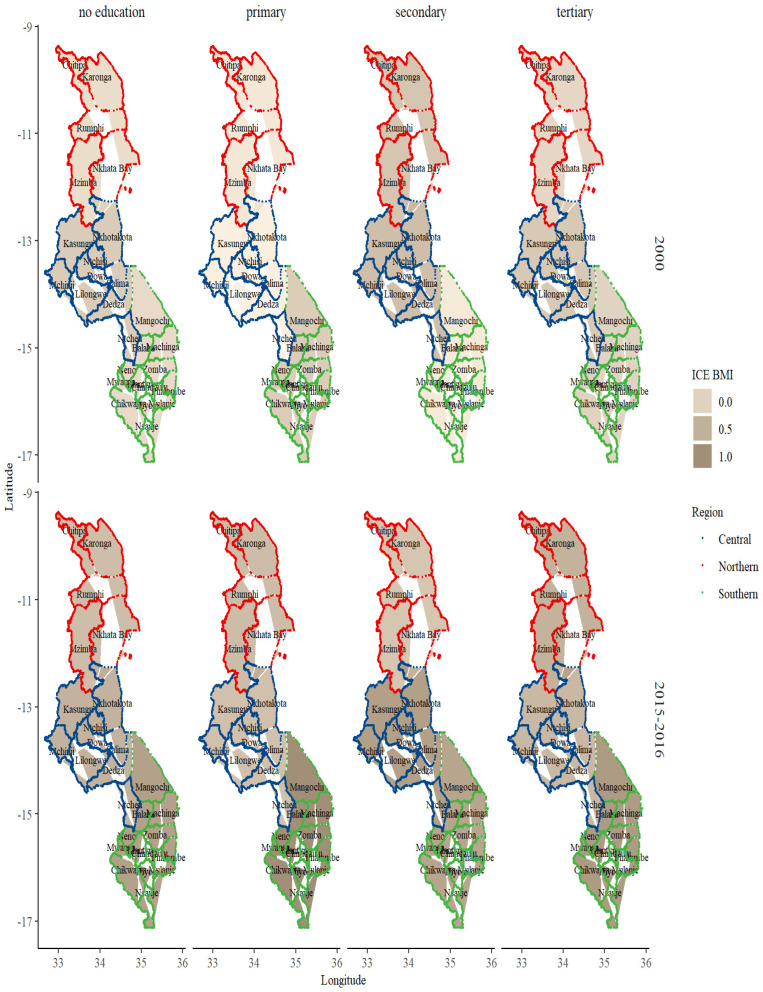

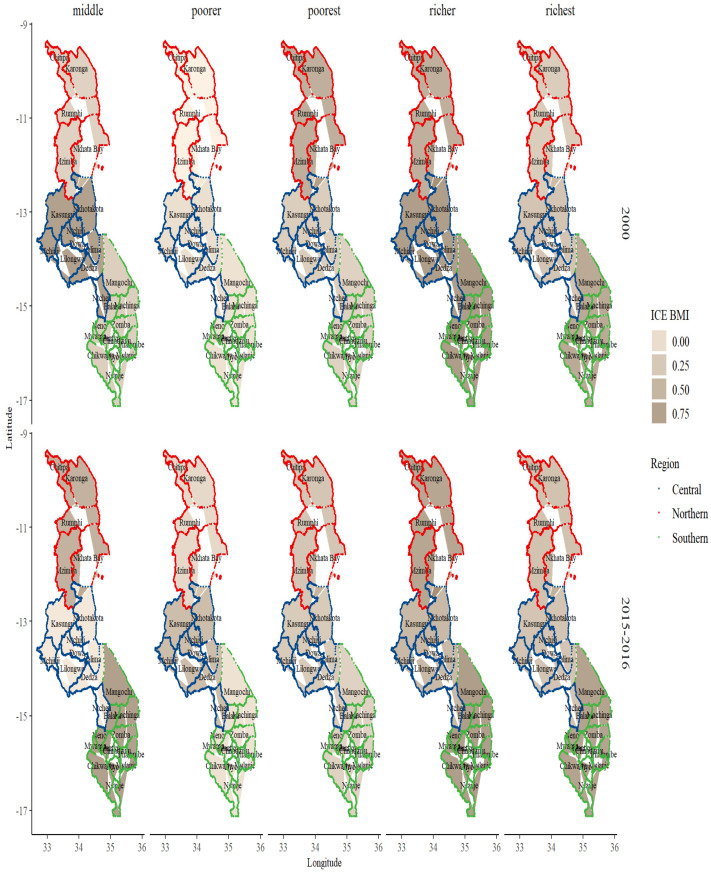
Concentration of extreme body mass index among females. Source: computed from MDHS 2000–2015–2016. Note: BMI: body mass index; red, blue, and green lines represent areas in the northern, central, and southern regions of Malawi.

Malawi is a landlocked country in south-eastern Africa. The country lies within 13°30′ South latitude and 34° East longitude and has an estimated land area of 118,484 km^2^ [40]. Malawi is divided into three administrative regions—North, Central, and South—with 28 subdivisions (districts) (see Figure 2 and Figure 3). The country has one of the highest parity, infant mortality [13,14,15], overweight, and obesity rates in SSA [6,8].

### 2.3. Study Variables

The study variables included sociodemographic profiles (i.e., age, residence, wealth quintile, level of education, and region/district), anthropometrics and health (i.e., pregnancy status, BMI), fertility, mortality (i.e., the total number of births, number of dead sons, number of dead daughters), GPS coordinates (longitudes and latitudes), and other information (year of interview, cluster number, and all DHS indicators used for constructing wealth index for Malawi in 2000 (see https://dhsprogram.com/topics/wealth-index/Wealth-Index-Construction.cfm accessed on 26 May 2021). Apart from BMI, all other variables were self-reported. These variables provided information on cross-national factors that affect and reflect health outcomes—low and high BMI and child mortality. In addition, these variables could be used to generate complex but accurate models [41,42].

Age, number of births, number of dead sons, number of dead daughters, and BMI were numeric variables. Wealth quintile groups, residence, regions/districts, and level of education were categorical variables. The wealth quintile groups were poorer, poor, middle, rich, and richest. Age was transformed into two different sets of categorical variables: (1) 15–24, 25–34, 35–49 and (2) abridged five-year cohorts (i.e., 15–19, 20–24, 25–29, 30–34, 35–39, 40–44, 45–49). The latter (abridged five-year cohorts) was introduced to support indirect mortality analysis. The former (15–24, 25–34, 35–49 age cohorts) reflected significant stages of aging [43,44]. Level of education was categorized as no education, primary, secondary, and tertiary. BMI was categorised as <18.5 kg/m^2^, underweight; 18.5–24.9 kg/m^2^, normal; 25.0–29.9 kg/m^2^, overweight; and ≥30.0 kg/m^2^, obese [45]. The classification of BMI into the above-identified groupings is under the recommended WHO standard cutoffs. The study did not use ethnic-specific BMI cutoffs recommended by other institutions and researchers as ethnic-specific BMI cutoffs serve as a backdrop for disease risk rather than a self-standing medical condition [46,47]. BMI and child mortality were the outcome variables for the study. Child mortality referred to the number of dead children, irrespective of age at death. All females who were pregnant at the time of each survey were omitted from the sample. The sum of dead daughters and sons per female became the total number of children dead per female or mother. Each female survey and the corresponding GPS data were integrated using the cluster number and year of interview variables. Missing values for the variables in each survey year were imputed using the classification and regression tree (cart) imputation approach to minimize the effects of missing data on estimates [48,49]. All four surveys were merged, and the study’s final sample was 65,499.

### 2.4. Data Analyses

Descriptive statistics were computed and ranked by year to provide a clearer understanding of the temporal distribution of the indicators in the Malawian population. The distribution was complemented with a test of variation in the outcome. In addition, average BMI per year was ranked by sociodemographic status. In addition, the concentration of underweight versus obesity per age, residence, level of education, and wealth quintile categories was estimated. The concentrations of these extreme BMI categories for all districts/regions in Malawi were mapped out for the initial year (2000) and final year (2015–2016) to assess the polarity of health over space and time [50]. The index of concentration of BMI at the extremes (ICE) was computed using the following formula:

$$ICE_{xi}=\frac{\left(H_{xi}-L_{xi}\right)}{T_{xi}}$$

*H_xi_* is the number of obese persons who were within an *x* sociodemographic group, while *L_xi_* is the number of underweight persons in that *x* sociodemographic group in the *i*th region/district. *T_i_* is the total population of the specific demographic group in the region/district [51,52]. The ICE estimates range from −1 to 1, where −1 and 1 correspond to complete low and high levels of BMI [51]. Indirect life table estimates were generated to analyze the link between females’ BMI and child mortality. In perspective, information on children ever born and dead was used to estimate the level of child mortality among underweight, normal, overweight, and obese females. The total number of females (Wi) in an age group (i), the reported number of children ever born (Bi) to females in an age group, the reported number of dead children to a female (Di) in an age group, the proportion of dead children to females (di) in a certain age group, and the average parity per female (Pi) in an age group were computed (see Table 1). Further, these indices were used to estimate child mortality multipliers (ki) and the probability of dying (q(x) and surviving (l[x]) among children.

ai, bi, and ci are West model coefficients for estimating mortality multipliers in an age group of a mother/female. $P_{1},P_{2},$ and $P_{3}$ are average parity per female in the first, second and third age groups [43]. The corresponding mortality levels $\left(ML_{WM}\right)$ for the survival probability of child born to females in an age group were computed using the West model l(x) levels [43,53]. All analyses were weighted and computed with R statistical software version 4.12, a software environment and programming language designed for statistical analysis, graphical representation, and reporting. The sf, dplyr, mice, survey, ggplot2, u5mr, and sp R packages were used for the analysis [54,55,56,57,58,59,60].

## 3. Results

### BMI and Sociodemographic Profile Distribution

Table 2 shows the distribution of sociodemographic profiles of 65,499 female Malawians from 2000 to 2015–2016. There was an increase in the population of overweight, obese, and rich females over time. Many females were in the southern regions of Malawi. Approximately 90.5% (10,560) of these females resided in the rural hinterlands. Urban residence was highest in 2015–2016 and lowest in 2000. The average number of dead children per mother was highest in 2000 and lowest in 2015–2016. In 2000, 11,663 females were sampled, and 5109 (43.8%) were aged 15–24 years. In addition, about a fifth (2413 [20.7%]) of all females were within the poorest wealth quintile group. Less than a quarter (12.8%) of these females were overweight (1190 [10.2%]) and obese (302 [2.6%]). In 2015–2016, a total of 22,729 were sampled. Within this cohort, 6095 (26.8%) were within the richest wealth quintile group, and a majority (9358 [41.2%]) of the respondents were in the youngest age group (15–24 years). About a quarter of these females were overweight (3459 [15.2%]) and obese (1668 [7.3%]) in 2015–2016.

The trends of average BMI among females within different sociodemographic groups are illustrated in Figure 2. There were variances in the average BMI among females in the five sociodemographic groups. There was also a decline in BMI for all demographics in 2015–2016 following the rapid increase in 2010. Obesity and overweight were highest among all age groups in 2010 and lowest among all age groups in 2000. The mean BMI estimate was highest among females aged 35–49 years. Rural residents (22.1 to 24.3) had, on average, a lower BMI than urban residents (23.3 to 25.5). Females with tertiary education had the highest mean BMI compared with those with no primary education. From 2000 to 2015–2016, the average BMI was highest among females in the rich wealth quintile group and lowest among females in the poorest wealth quintile group.

The ICE measurement outcome for BMI across different sociodemographic groups in 2000 and 2015–2016 is shown in Figure 3. Generally, there was an uneven concentration of BMI over space and time in Malawi. In 2000, the concentration of obesity among many females was less prevalent as compared to 2015–2016. The concentration of BMI (i.e., in the direction of more concentration of obesity) was greater in the southern and central regions and lower (i.e., in the direction of more concentration of underweight) in the northern region. In 2015–2016, the concentration of obesity was higher among females who were older (35–49 years), urban residents, richest, richer, at least were primary school graduates, and located in districts within the southern and central regions. In contrast, more underweight cases were recorded in the northern region and among females in the poorest wealth quintile group.

Table 3 illustrates females’ child mortality experiences in 2000 and 2015–2016. Again, there were time and age group differentials in the child mortality estimates. Child mortality was highest among teenagers/adolescents and young people (15–19 and 20–24 years) and lowest among older (40–44 and 45–49) females. The mortality estimates were higher in 2015–2016 and lower in 2000. However, for all years and all population groups, the mortality estimates showed a steady decline as the age of females/mothers advanced. Among the different BMI groups, child mortality was highest among females who were underweight, overweight, and obese and lowest among females who were normal.

## 4. Discussion

The current study examined BMI and child mortality among females in Malawi. The concentration of BMI and child mortality were assumed to be best captured at the macro and micro levels by considering spatial and anthropometric variances. In so doing, we applied for the first time, the index of concentration at the extremes and indirect life table estimation techniques, which permitted the estimation of extreme BMI concentration at the regional/district level and child mortality among females in different BMI groups.

From 2000 to 2015–2016, the populations of obese and overweight females increased from 2.6% to 7.3% and 10.2% to 15.2%, respectively. This finding affirms previous research reports on the doubling of obesity among females in Malawi and other Sub-Saharan African countries [26,61]. The rise in obesity and overweight was, however, highest in 2010. This finding has been linked with sustained mild food and nutrition-related shocks from 2005 to 2010 and the trend of BMI increase. Under this pretext, the lower levels of obesity and overweight might be period effects and severe food and nutrition-related shocks—in 2015, Malawi was badly affected by flooding and drought that crippled crop production and food security in the same period [62,63]. The trend in BMI increase reinforces the need for a supportive environment and significant public policy reforms targeting lifestyles to curtail the rising obesity cases. Further, engagement of the food and health industries in the creation of obesity control programs will be a significant step in fighting the growing epidemics of obesity in Malawi and SSA in general. Besides the conventional behavioral factors that may have contributed to such experiences [16], the doubling of obesity among these females may be anthropogenic effects (females’ physiology), as females have more fat than lean mass regional deposition [4,23]. The unique female physiology signals the need for health metrics that can accurately detect Malawians at risk of obesity.

Many female Malawians were within the youthful age group (15–24 years), a feature common in SSA. The similarity between the age distribution in Malawi and other African countries has been attributed to factors ranging from high fertility [64,65] and overall demographic transition to recent advances in health care [66,67]. Moreover, the population is youthful because there is a gradual decline in the number of young people entering adulthood [68,69]. Adolescents and young are vulnerable to community and household influences that increase avoidable mortality risk [70,71]. The increased susceptibility to mortality during adolescence might reduce the number of adolescents and young people entering adulthood. Therefore, a critical assessment of the unmet needs of young people is needed to determine the most appropriate youth-specific health interventions focusing on preventable deaths.

Across the ICE measures, obesity was highly concentrated among females in the central and southern regions, older (35–49 years), urban residents, educated (tertiary), and rich. However, areas in the northern region had the lowest concentration of obesity. These spatial outcomes may represent many distal and surrogate conditions in areas and how those conditions shape health (BMI) [72]. The higher prevalence of obesity in the areas in the central and southern regions may be a population effect—densely populated areas are likely to have high proportions of obese persons [20]. Additionally, residents may quickly form unhealthy behaviors (i.e., dietary and physical activity) which, in turn, increase obesity risk [18,19,25]. Moreover, as wealth continues to soar in these areas, there is a trickle-down effect—improvement in socioeconomic status in neighboring areas such as Dedza, Chiradzulu, Phalome, and Dowa further shifts obesity risk to females in areas with less wealth [18,19,26,73,74]. Contrarily, the concentration of lower levels of obesity in the northern region (i.e., areas such as Karonga and Chitipa) might be related to the higher prevalence of stunting during childhood through to adulthood [75,76].

The decrease in child mortality among females in older years than their counterparts in the young ages in Malawi is reminiscent of SSA and other LMICs [65,77]. In addition, there were polarized child mortality estimates as females on the left (i.e., underweight) and right (i.e., overweight and obese) ends of the BMI spectrum than those in the middle (normal) recorded higher rates. Felisbino-Mendes et al. [78] identified similar results in Brazil, where females with high BMI were more likely to experience the death of a child. The main reasons for such mortality experiences among underweight, overweight, and obese female Malawians are unknown. However, contagion and competition, biological insufficiency, and maternal depletion factors could, in part, explain the current observation [79]. Drawing from Bean et al. [79], the higher prevalence of child death among young mothers than among mature mothers could be due to biological insufficiency. Young females, unlike older females, are biologically immature for bearing and raising children because of their incomplete anatomical and physiological development. Such biological deficiencies account for obstetric disorders and associated incidence of child death [80]. Another pathway is that poor maternal outcomes are associated with unhealthy anthropogenic dispositions and perilous medical conditions. The preponderance of low BMI (underweight) is greater among young than older females, and it is known that underweight females are often vulnerable to medical conditions such as anemia, eclampsia, infections, and puerperal endometritis. These health conditions may cause poorer birth outcomes, including low birth weight, increased disease risk, and child death [81,82,83]. Thus, the increased risk of child death among younger and underweight females may be related to their biology. This linkage signals the need to prevent teenage pregnancy and promote health programs targeting iron deficiency and unmet reproductive health needs among young people. A plausible maternal depletion outlook is that multiparous females might have poor health and birth outcomes. Similarly, multiparous females are often older and obese and overweight. The BMI status of such females may be due to the repeated gestational and postnatal weight gain [84,85]. Multiparity often has deleterious effects on mothers’ health as mothers may not recover hormonally, emotionally, and nutritionally [86]. Multiparity may increase the risk of poor maternal outcomes, including the death of infants and children in general [79,87]. The higher levels of child mortality among females who were overweight and obese may be due to low parental investment and competition for resources. In resource-limited and high-parity areas such as Malawi, there is the possibility of siblings’ competition for finite resources that puts younger children or newborns at a disadvantage. Similarly, the intensity of scarce resources may further be influenced by the mother’s inactivity, a common practice among obese females [88,89,90]. Where young children or infants have less access to resources, even at the subsistence level, they become malnourished—lacking the right amount of nutrients needed for survival—and susceptible to infectious diseases, the leading causes of death among children in Malawi and SSA [71,91]. Moreover, the persistence of poor health care access for the marginalized groups, including obese females and children in Malawi [92,93], further increases the mortality risk among children born to obese and overweight females.

### 4.1. Strength and Limitations

This study is the first to examine anthropometric disposition, parity, and mortality in Malawi using micro- and macro-level indicators, ICE, and indirect demographic techniques. This study provides useful spatial information that warrants the use of ICE as a metric for assessing disparities in health at the macro level and further suggests the appropriate context-specific interventions in Malawi. In addition, the present findings are generalizable to non-pregnant females in the country. Despite these strengths, the findings should be interpreted with caution. First, although BMI is an anthropometric indicator of adiposity, it does not differentiate between body fat and lean body mass, nor does it incorporate age differentials. Second, computations of the study are based on cross-sectional rather than longitudinal or age cohort studies.

### 4.2. Practical Implications

Reporting estimates of child mortality and regional-level variations of BMI among females over time is essential for a clearer understanding of the determinants of health in Malawi. Sociodemographic and context or compositional differences across regions provide insights into the required and beneficial strategies to be implemented to reduce the prevalence and burden of malnutrition (i.e., underweight, overweight, and obesity). In addition, among the health-related aspirations of the sustainable development goals (SDGs), is the reduction of mortality. Thus, if the relationship between malnutrition (i.e., underweight, overweight, and obesity) and child mortality is left unchecked, efforts to rapidly reduce mortality in Malawi will be hampered. Therefore, devoting considerable efforts toward the continuous assessment of the magnitude of BMI differences per area demographics would give possible clues on the role these distal factors play on health. Such clues could also guide future research on the etiological (i.e., genetic and environmental) predictors accounting for the risk of overweight, obesity, and child mortality in Malawi and perhaps elsewhere in the sub-region.

## 5. Conclusions

The present study investigated socioeconomic health inequality, using BMI as the main health indicator, and the association between BMI and child mortality among females in Malawi. National health and vital statistics are needed for all population groups in Malawi. However, while national-, regional-, and individual-level statistics and reports are in development, we provide these results to inform health experts and other stakeholders about where and how programs and interventions are needed most. Although under-five and infant mortality, as well as the epidemics of malnutrition (i.e., underweight, overweight, and obesity), have been reported and investigated, the findings suggest that along with the existing interventions (e.g., health campaigns), high-quality child mortality and region/district-specific extreme BMI estimates can assist Malawi and the international community in accelerating health promotion efforts. These health promotion efforts could be geographically targeted programs to reduce the risk of obesity among females in regions with high prevalence.

## Figures and Tables

**Table 1:** Formulae for derived measures.

Index
di=Di/Bi
Pi=Bi/Wi
$ki=ai+bi*\left(\frac{P_{1}}{P_{2}}\right)+ci*\left(\frac{P_{2}}{P_{3}}\right)$
q(x)=ki∗di
l(x)=1−q(x)
$ML_{WM}=\frac{l\left(x\right)-l{\left(x\right)}_{a}}{l{\left(x\right)}_{b}-l{\left(x\right)}_{a}}$

Source: adapted from Preston et al. (2000) [43].

**Table 2:** Sociodemographic profiles of females in Malawi from 2000 to 2015–2016.

	Period of Study				Total	
	2000 (*n* = 11,663)	2004–2005 (*n* = 10,249)	2010 (*n* = 20,858)	2015–2016 (*n* = 22,729)	(*n* = 65,499)	*p* Value
	Information on Categorical Indicators					
	(100%)	(100%)	(100%)	(100%)	(100%)	
Age Groups						<0.001
15–24	5109 (43.8%)	4432 (43.2%)	8442 (40.5%)	9358 (41.2%)	27,341 (41.7%)	
25–34	3378 (29.0%)	3132 (30.6%)	6733 (32.3%)	6986 (30.7%)	20,229 (30.9%)	
35–49	3176 (27.2%)	2685 (26.2%)	5683 (27.2%)	6385 (28.1%)	17,929 (27.4%)	
Residence						<0.001
Urban	1103 (9.5%)	1469 (14.3%)	2884 (13.8%)	4921 (21.7%)	10,377 (15.8%)	
Rural	10,560 (90.5%)	8780 (85.7%)	17,974 (86.2%)	17,808 (78.3%)	55,122 (84.2%)	
Education						0.02
No education	9124 (78.2%)	7717 (75.3%)	14,908 (71.5%)	14,532 (63.9%)	46,281 (70.7%)	
Primary	2015 (17.3%)	1975 (19.3%)	4549 (21.8%)	5964 (26.2%)	14,503 (22.1%)	
Secondary	506 (4.3%)	493 (4.8%)	1098 (5.3%)	1588 (7.0%)	3685 (5.6%)	
Tertiary	18 (0.2%)	64 (0.6%)	303 (1.5%)	645 (2.8%)	1030 (1.6%)	
Wealth Quintile						<0.001
Poorest	2413 (20.7%)	1834 (17.9%)	4071 (19.5%)	3889 (17.1%)	12,207 (18.6%)	
Poorer	2350 (20.1%)	2003 (19.5%)	4008 (19.2%)	4037 (17.8%)	12,398 (18.9%)	
Middle	2569 (22.0%)	2152 (21.0%)	4192 (20.1%)	4170 (18.3%)	13,083 (20.0%)	
Richer	2395 (20.5%)	2135 (20.8%)	4317 (20.7%)	4538 (20.0%)	13,385 (20.4%)	
Richest	1936 (16.6%)	2125 (20.7%)	4270 (20.5%)	6095 (26.8%)	14,426 (22.0%)	
Region						0.01
North	1925 (16.5%)	1410 (13.8%)	3794 (18.2%)	4440 (19.5%)	11,569 (17.7%)	
Central	3932 (33.7%)	3656 (35.7%)	7117 (34.1%)	7773 (34.2%)	22,478 (34.3%)	
South	5806 (49.8%)	5183 (50.6%)	9947 (47.7%)	10,516 (46.3%)	31,452 (48.0%)	
BMI Categories						<0.001
Underweight	1049 (9.0%)	948 (9.2%)	1658 (7.9%)	1689 (7.4%)	5344 (8.2%)	
Normal	9122 (78.2%)	7942 (77.5%)	14,597 (70.0%)	15,913 (70.0%)	47,574 (72.6%)	
Overweight	1190 (10.2%)	1097 (10.7%)	2886 (13.8%)	3459 (15.2%)	8632 (13.2%)	
Obese	302 (2.6%)	262 (2.6%)	1717 (8.2%)	1668 (7.3%)	3949 (6.0%)	
	Information on Numerical Indicators					
Number of Children Ever Born						<0.001
Mean (SD)	3.13 (2.89)	3.17 (2.77)	3.19 (2.76)	2.84 (2.48)	3.06 (2.70)	
Range	0.00–16.00	0.00–16.00	0.00–17.00	0.00–15.00	0.000–17.00	
Number of Children Dead to a Mother/Female						<0.001
Mean (SD)	0.68 (1.17)	0.57 (1.05)	0.50 (0.96)	0.30 (0.73)	0.47 (0.96)	
Range	0.00–10.00	0.00–11.00	0.00–11.00	0.00–9.00	0.00–11.00	

Source: computed from MDHS 2000, 2004–2005, 2010, 2015–2016. Note: *n* denotes number of observations; *p* < 0.05, *p* < 0.01, *p* < 0.001 *t* test statistical significance; BMI: body mass index.

**Table 3:** Estimates of child mortality via parity and child survival among females.

Mortality Levels								
			Body Mass Index (BMI) Categories					
Indicator	Total Population		Underweight		Normal		Overweight and Obese	
Mother’s Age	2000	2015–2016	2000	2015–2016	2000	2015–2016	2000	2015–2016
15–19	12.83	17.48	14.03	19.87	13.33	17.96	12.1	19.58
20–24	12.99	19.45	12.61	18.67	13.27	20.04	13.59	20.24
25–29	12.03	18.96	12.31	18.55	11.85	19.32	13.55	18.69
30–34	12.72	17.38	12.05	18.52	12.68	18.2	12.51	18.19
35–39	12.12	16.74	11	17.22	12.04	17.16	13.22	17.71
40–44	11.8	15.75	10.36	15.27	11.42	16	12.76	16.43
45–49	10.85	14.77	10.14	15.05	10.53	15.41	13.1	15.87

Source: computed from MDHS 2000 and 2015–2016. Note: BMI: body mass index.

## Data Availability

The data used for the study are available at: https://dhsprogram.com/data/dataset_admin/index.cfm, accessed on 26 May 2021.

## Abbreviations

BMI	Body Mass Index
DHS	Demographic and Health Survey
MDHS	Malawi Demographic and Health Survey
GPS	Global Positioning System
ICE	Index of Concentration at the Extremes
LMICs	Low- and Middle-Income Countries
PSUs	Primary Sampling Unit
SEAs	Standard Enumeration Areas
SSA	Sub-Saharan Africa
SDGs	Sustainable Development Goals
USAID	United States Agency for International Development
UNICEF	United Nations Children’s Fund
WHO	World Health Organisation
